# Disruption of the sialic acid/Siglec-9 axis improves antibody-mediated neutrophil cytotoxicity towards tumor cells

**DOI:** 10.3389/fimmu.2023.1178817

**Published:** 2023-06-06

**Authors:** Marta Lustig, Chilam Chan, J. H. Marco Jansen, Maria Bräutigam, Max A. Kölling, Carina Lynn Gehlert, Niklas Baumann, Simone Mester, Stian Foss, Jan Terje Andersen, Lorenz Bastian, Peter Sondermann, Matthias Peipp, Renate Burger, Jeanette H. W. Leusen, Thomas Valerius

**Affiliations:** ^1^ Division of Stem Cell Transplantation and Immunotherapy, Department of Medicine II, Christian-Albrechts-University Kiel and University Medical Center Schleswig-Holstein Campus Kiel, Kiel, Germany; ^2^ Center for Translational Immunology, University Medical Center Utrecht, Utrecht, Netherlands; ^3^ Tacalyx GmbH, Berlin, Germany; ^4^ Division of Antibody-Based Immunotherapy, Department of Medicine II, Christian-Albrechts-University Kiel and University Medical Center Schleswig-Holstein Campus Kiel, Kiel, Germany; ^5^ Institute for Clinical Medicine, Department of Pharmacology, University of Oslo and Oslo University Hospital, Oslo, Norway; ^6^ Institute for Clinical Medicine, Department of Immunology, University of Oslo and Oslo University Hospital, Oslo, Norway; ^7^ Department of Medicine II, Christian-Albrechts-University Kiel and University Medical Center Schleswig-Holstein, Kiel, Germany

**Keywords:** sialic acid, Siglec-9, neutrophils (PMNs), ADCC - antibody dependent cellular cytotoxicity, glycans, myeloid cells, siglecs

## Abstract

Upregulation of surface expressed sialoglycans on tumor cells is one of the mechanisms which promote tumor growth and progression. Specifically, the interactions of sialic acids with sialic acid-binding immunoglobulin-like lectins (Siglecs) on lymphoid or myeloid cells transmit inhibitory signals and lead to suppression of anti-tumor responses. Here, we show that neutrophils express among others Siglec-9, and that EGFR and HER2 positive breast tumor cells express ligands for Siglec-9. Treatment of tumor cells with neuraminidases or a sialyl transferase inhibitor significantly reduced binding of a soluble recombinant Siglec-9-Fc fusion protein, while EGFR and HER2 expression remained unchanged. Importantly, the cytotoxic activity of neutrophils driven by therapeutic EGFR or HER2 antibodies *in vitro* was increased by blocking the sialic acid/Siglec interaction, either by reducing tumor cell sialylation or by a Siglec-9 blocking antibody containing an effector silenced Fc domain. *In vivo* a short-term xenograft mouse model confirmed the improved therapeutic efficacy of EGFR antibodies against sialic acid depleted, by a sialyltransferase inhibitor, tumor cells compared to untreated cells. Our studies demonstrate that sialic acid/Siglec interactions between tumor cells and myeloid cells can impair antibody dependent tumor cell killing, and that Siglec-9 on polymorphonuclear cells (PMN) is critically involved. Considering that PMN are often a highly abundant cell population in the tumor microenvironment, Siglec-9 constitutes a promising target for myeloid checkpoint blockade to improve antibody-based tumor immunotherapy.

## Graphical Abstract

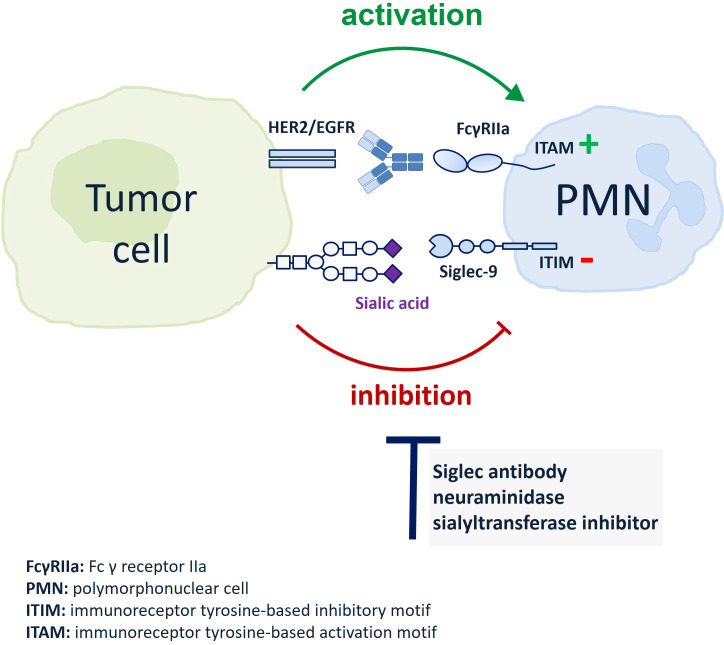

## Introduction

The outer membrane of cells is typically covered with a dense coat of sugars called glycocalyx (1). The glycocalyx constitutes a layer of polysaccharides that are covalently bound to membrane expressed non-carbohydrate molecules such as lipids and proteins. Monosaccharides are the basic structural units of glycans linked together via different glycosidic bonds, providing a broad diversity of glycans with modulatory and recognition functions (1–4). Alterations in the glycocalyx are often linked with diseases, including cancer where they can be associated with poor patients’ prognosis (3, 5, 6). Among the various glycosylation patterns, upregulation of cell surface sialoglycans is one of the common mechanisms which favours tumor growth and progression (7). Enhanced activity of sialyltransferases or decreased neuraminidase activity are observed as underlying causes of hypersialylation (8–10). In cancer, these and other glycocalyx alterations can also lead to the expression of so called tumor associated carbohydrate antigens (TACAs), that can constitute targets for specific tumor immunotherapy against many tumor types, including breast cancer (10, 11).

In tumor development, sialic acids regulate immune evasion e.g. by engaging inhibitory Siglec receptors on myeloid and other immune cells. Siglecs constitute a family of immune-modulatory receptors that belong to the I-type immunoglobulin (Ig)-like lectin family (3, 12, 13). Siglecs are divided into two groups based on their sequence similarity and evolutionary conservation. Interestingly, a subset of distantly related Siglecs seems to be conserved in mammals (sialoadhesin/Siglec-1, CD22/Siglec-2, MAG/Siglec-4 and Siglec-15). In comparison, the subset of CD33-related Siglecs consists of eleven human Siglecs encoded by genes on chromosome 19q, which share 50% - 99% identity in man, but are divergent between species. Nine of the CD33 related Siglecs constitute inhibitory Siglecs, which have immunoreceptor tyrosine-based inhibitory motifs (ITIM) in their intracellular domain. ITIMs become phosphorylated upon ligand binding, recruit tyrosine phosphatases like SHP-1 and SHP-2 and ultimately trigger immunosuppressive signaling (3, 4, 12, 13).

In the immune suppressive tumor microenvironment, it has been reported that Siglecs expression is increased in tumor-infiltrating immune cells and sialic acid expression enhanced on diverse tumor types, resulting in the modulation of multiple immune cells activation by Siglecs (11, 14). For example, sialic acid/Siglec interactions have been shown to favour an immunosuppressive tumor microenvironment, while sialic acid blockade promotes T and natural killer (NK) cell infiltration resulting in tumor growth reduction (15, 16). Tumor sialoglycans interfere with cytotoxic T and NK cell functions by preventing their killing mechanisms through the engagement of Siglec-9 and Siglec-7, respectively (17–20). Furthermore, blockade of CD24 interaction with Siglec-10, expressed by tumor-associated macrophages caused enhancement of phagocytic activity and reduction of tumor growth (21). Recently, disruption of the disialoganglioside GD2/Siglec-7 axis with a GD2 antibody in combination with a CD47 antibody resulted in tumor eradication in syngeneic and xenograft mouse models of neuroblastoma by recruitment of macrophages (22). Antibodies against Siglec-9 and Siglec-7 reduced the tumor cell burden in different *in vivo* models by interfering with myeloid cells in Siglec-9/Siglec-7 double transgenic immunocompetent mice (23–25). Targeting glycans on tumor cells is another approach to interfere with sialic acid/Siglec interactions. Reducing the glycan content with a therapeutic antibody coupled to neuraminidase enhanced myeloid cell mediated anti-tumor responses *in vivo* (26). Regarding neutrophil activation, Siglec-9 was shown to reduce their cytotoxic activity against tumor cells *in vitro* and *in vivo*, and polymorphisms of Siglec-9 that diminish ligand binding have been shown to provide better survival in non-small cell lung tumor patients (18, 27). While there is sufficient evidence that interactions with Siglecs represent an important immune checkpoint for T cells, NK cells and macrophages (17–19, 21, 28), the extent to which tumor sialoglycans regulate neutrophil activation is less explored.

Polymorphonuclear cells are the most abundant leukocyte population in the circulation and are present in many tumor cell infiltrates, where they often have immunosuppressive functions (29). However, PMN also have potential killing activity against tumor cells (30), suggesting that they can become a promising effector cell population upon proper engagement. For example, antibody-dependent cytotoxicity of PMN was significantly enhanced when IgA or IgG2 rather than IgG1 antibodies against tumor associated antigens (TAA) were employed (31–33). However, currently most clinically approved antibodies for tumor therapy are of the IgG1 isotype. IgG antibodies are powerful tools against tumors as they effectively trigger antibody-dependent cellular cytotoxicity (ADCC) by NK cells, antibody-dependent cellular phagocytosis (ADCP) by macrophages, induce complement-dependent cytotoxicity (CDC) and also have a long plasma half-life. However, PMN often cannot efficiently be engaged by human IgG1 antibodies, because they abundantly express the GPI-linked FcγRIIIb isoform, which binds human IgG1 antibodies, particularly when they are engineered for increased FcγRIII-binding affinity, but does not trigger ADCC (34, 35).

Over the last decade, immune checkpoint inhibition has revolutionized tumor therapy (36, 37). In addition to T cell immune checkpoints, also innate myeloid checkpoint blocking molecules are moving into clinical development. Currently the clinically most advanced agents targeting myeloid checkpoints block the interaction between CD47 on tumor cells and the signal regulatory protein alpha (SIRPα) expressed on myeloid cells (38, 39). For example, the combination of the CD47 blocking antibody magrolimab with rituximab demonstrated clinical activity in patients with non-Hodgkin’s lymphomas and was the first clinical study to confirm the scientific rationale for myeloid immune checkpoint blockade in tumor patients (40).

To improve therapeutic antibody mediated tumor cell killing by PMN, novel immune checkpoints need to be identified. Here, we investigated the roles of tumor cell expressed sialic acid on the one hand and Siglec-9 on PMN on the other hand. We employed antibodies of IgG1 or IgG2 isotypes against the tumor associated antigens EGFR and HER2 to demonstrate that sialic acid reduction on breast carcinoma cells enhanced ADCC activity mediated by PMN. Importantly, our results show that Siglec-9 is involved as an inhibitory receptor in PMN mediated tumor cell killing.

## Materials and methods

### Cell lines and culture

The human breast cancer cell lines MDA-MB-468 and SK-BR-3 were used as target cells for EGFR or HER-2 antibodies, respectively. All cell lines were obtained from the DSMZ (German Collection of Microorganisms and Cell Cultures, Braunschweig, DE) and were grown in media as recommended. Cells were regularly monitored for *Mycoplasma* infection using PCR-based methods. Cells were cultured in a humidified 5% CO_2_ atmosphere at 37°C.

### Monoclonal antibodies

The clinically approved antibodies cetuximab (chimeric human IgG1; clone 225, Erbitux^®^), panitumumab (human IgG2; clone E7.6.3, Vectibix^®^) and trastuzumab (humanized IgG1; clone 4D5-8; Herceptin^®^) were obtained from Merck (Darmstadt, DE), Amgen (Thousand Oaks, California, USA) and Roche (Basel, CH), respectively. The recombinant IgG2 variant of trastuzumab was expressed transiently from the pFUSE vector system (Invivogen) in Expi293 cells (Thermo Fisher). Pure monomeric fractions were obtained by affinity purification (CaptureSelect CH1) (Thermo Fisher) and size exclusion chromatography (SEC) (Superdex 200 Increase) (Cytiva Lifesciences) using an ÄKTA Avant 25 instrument.

### Removal of cell surface sialic acid

Cell surface sialic acid on tumor cells was removed either enzymatically by treatment (1 h, 37 °C, PBS as control) with neuraminidases from Vibrio cholerae (NEU-VC) (0.1 U/ml) or Clostridium perfringens (NEU-CP) (1 U/ml) (Roche, Basel, CH), or by inhibition of sialyltransferases with P-3Fax-Neu5Ac (STi) (MerckMillipore) (100 µM dissolved in DMSO) for 72 h, 37°C.

### Immunofluorescence

Immunofluorescence analyses to investigate monoclonal antibodies binding were performed on a Navios flow cytometer and analysed with Kaluza software (Beckman Coulter). For antibody details please refer to the Online **Supplementary Appendix**. Microscopic images (20 x magnifications) of Calcein-AM (10µM, for 30 min at 37°C), labelled tumor cells were taken using the fluorescence microscope Keyence BZ-X800 series (Ōsaka, JPN).

### Isolation of human effector cells and ADCC assay

In line with the ethical approval of the University of Kiel, human PMN were isolated as previously described by density gradient centrifugation from the peripheral blood of healthy donors after informed consent (35). ADCC activity was analysed in 3 hour (h) 51 chromium (^51^Cr) release assays using GM-CSF (50 U/ml, CellGenix GmbH) *in vitro* stimulated PMN at an effector-to-target cell (E:T) ratio of 40:1, as previously described (35).

### Production and purification of a Siglec-9 IgG2 blocking antibody

Variable light (VL) and heavy chain (VH) sequences of the human Siglec-9 blocking antibody (clone mAbA) derived from published sequences (24) were ligated into the expression vector pSEC-LC-kappa and pSec-HC-IgG2σ, respectively. The pSec-HC-IgG2σ vector encodes an IgG2σ, carrying a modified Fc domain to prevent FcγR and C1q binding (V234A/G237A/P238S/H268A/V309L/A330S/P331S) (41). Antibody production is described in the Online **Supplementary Appendix**.

### SDS-PAGE

Purified antibodies were separated by SDS-PAGE under non-reducing and reducing conditions using a 4-15% precast polyacrylamide gel (Mini-PROTEAN^®^ TGX™, BioRad). After a running time of 90 min at constant 120 V, gels were stained with Coomassie brilliant blue staining solution (Carl Roth, Karlsruhe, DE).

### *In vivo* experiment

Mice were maintained in the Central Laboratory Animal Research Facility of the University of Utrecht, NL. SCID mice (CB17/lcr-Prkdcscid/lcrlcoCrl; Charles River) were housed in groups in a temperature and 12:12 hour light:dark cycle controlled room, with food and water available ad libitum. The animal experiment was approved by the Animal Ethical Committee of the UMC Utrecht. MBA-MD-468 cells were treated with 100 μM STi or DMSO for 3 days and then labelled (15 min) with 4 μM (high) CellTrace Violet (CTV) fluorescent or 4 μM carboxyfluorescein succinimidyl ester (CSFE) dye (Invitrogen, Thermo Fisher Scientific). EGFR negative murine Ba/F3 control cells were labelled with 0.125 μM CTV (low) fluorescent dye. Cells were mixed at a 5:5:1 ratio (MDA-MB-468 DMSO : MDA-MB-468 STi : Ba/F3), and a total of 6 × 10^6^ cells were injected intraperitoneally into each mouse. Immediately after tumor cell inoculation, a single dose of 10 μg cetuximab, panitumumab, or the respective isotype controls were given by intraperitoneal injection; PBS was used as a control. Sixteen hours later, mice (n = 9 per group) were killed, and tumor cells recovered by peritoneal lavage using PBS containing 5 mmol/L EDTA. The ratio of MDA-MD-468 STi or MDA-MB-468 DMSO to Ba/F3 cells was determined by flow cytometry.

### Glycan array

N-hydroxysuccinimide (NHS) activated glass slides (CodeLink, SurModics) were used to immobilize glycans with a primary amine functionalization. Glycans (0.1 mM) were dissolved in sodium phosphate buffer (50 mM, pH 8.5) for the spotting. 64 identical arrays were printed onto each slide using a piezoelectric spotting device (Scienion). The glass slides were incubated overnight at room temperature in a moist chamber to complete the coupling via NHS, before they were treated with ethanolamine (50 mM) in sodium phosphate buffer (50 mM, pH 9) for 1 h at room temperature. After the remaining NHS groups were quenched, the slides were washed with H_2_O (3x) and then centrifuged to dryness (300 g, 5min). For Siglec analysis the microarray slide was blocked for 1 h at room temperature using BSA (3% in PBS), washed twice with PBS and once with H_2_O. After drying via centrifugation the slide was placed into a FlexWell 64 grid. The slide was incubated with the Siglec-9-Fc and the reference antibody 5B1 (murine CA19-9 antibody) (diluted with 3% BSA in PBS) for 1 h at room temperature in a moist chamber. The wells were washed with 0.1% Tween-20 in PBS (2x). The secondary goat anti-human IgG Fc-AF647 (SouthernBiotech) was diluted 1:200 and the goat anti-mouse IgG Fc–F4143 (Sigma Aldrich) 1:400 in 3% BSA in PBS and distributed into the wells. After 1 h of incubation in a moist chamber at room temperature, the wells were washed with 0.1% Tween-20 in PBS (2x). The grid was removed and the whole slide was washed with PBS (2x) and H_2_O (2x) before the slide was dried by centrifugation. The slide was scanned using an InnoScan 1100 AL (Synopsys). Background-subtracted fluorescence intensity (RFI) values were exported for further analysis.

### Statistical analysis

At least three independent experiments were performed for flow cytometric analysis and in triplicates for each ADCC assay with effector cells from different donors. Data were statistically analysed with GraphPad Prism Software using appropriate tests (San Diego, CA, USA). Significance was accepted with p < 0.05.

## Results

### α2,3 linked sialic acid on tumor cells can be reduced by neuraminidase treatment or sialyltransferase inhibition

**Figure 1:**
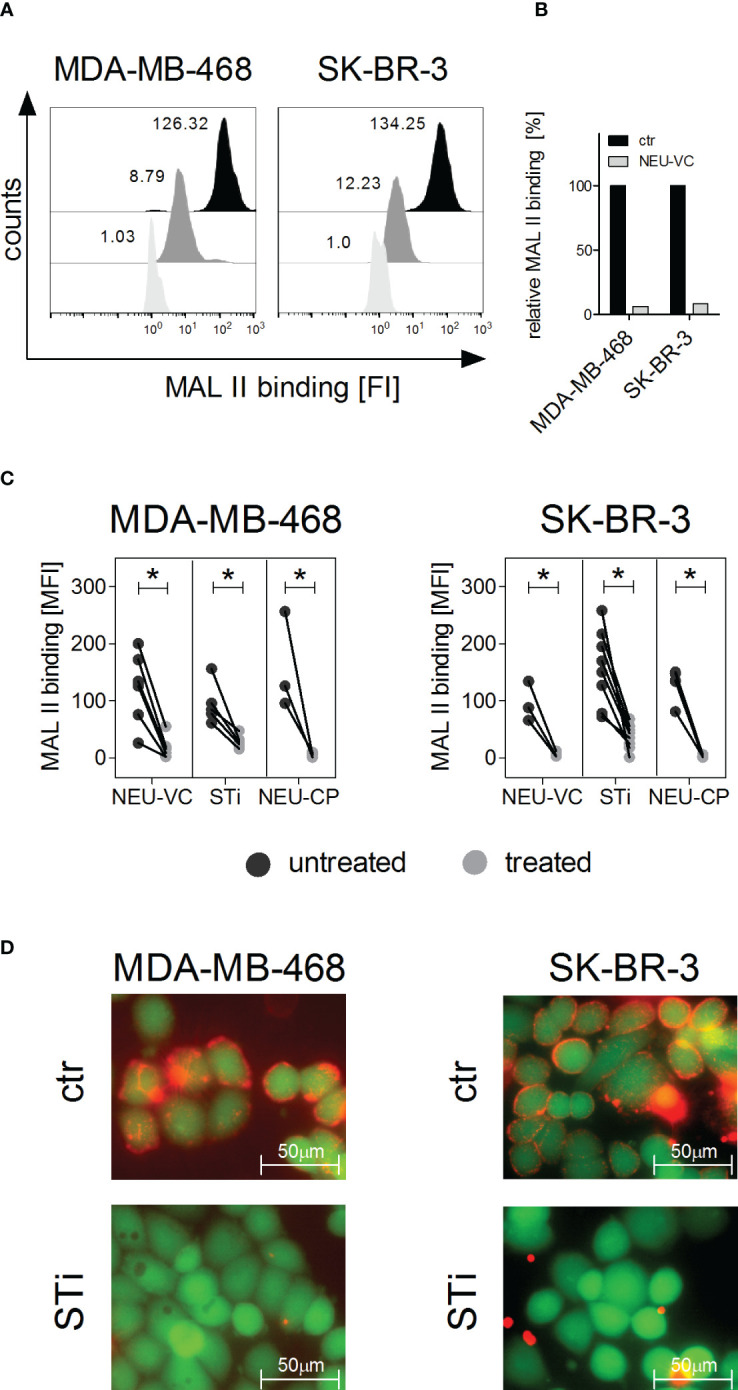
α2,3 linked sialic acid on tumor cells can be reduced by neuraminidase treatment or by sialyltransferase inhibition. **(A, B)** Binding of Maackia amurensis leukagglutinin (MAL) II (5 µg/ml), detected with streptavidin-PE, on MDA-MB-468 and SK-BR-3 cells is reduced after treatment with neuraminidase of Vibrio cholerae (NEU-VC) (0.1 U/ml). Black, untreated cells; grey, treated cells; white, PBS was used as control for MAL II on untreated cells. Shown are representative histograms **(A)** and MAL II binding normalized to the control (ctr) **(B)**. **(C)** MAL II binding on MDA-MB-468 and SK-BR-3 cells untreated (black dots) or treated (grey dots) with NEU-VC (0.1 U/ml), NEU-Clostridium perfringens (CP) (1 U/ml), or a sialyltransferase inhibitor (STi) (100 µM). Dot plots show MAL II binding as mean fluorescence intensity (MFI) values. * depicts significant differences (p < 0.05) between treated and untreated cells (two-way ANOVA). FI, fluorescence intensity. **(D)** Overlay of microscopic images of live (green fluorescent, excitation at 495nm) and sialic acid positive (red fluorescent, excitation at 532 nm) MDA-MB-468 and SK-BR-3 cells. Untreated (ctr) or STi treated cells (STi) were stained with Calcein-AM (green fluorescent) and MAL II-streptavidin-PE (red fluorescent).

The EGFR or HER2 positive breast cancer cell lines MDA-MB-468 and SK-BR-3, respectively, were chosen as model systems to evaluate the role of sialic acid on tumor cell killing by PMN. To assess the presence of sialic acid on the cell surface of the two cell lines, binding of the lectin Maackia amurensis leukoagglutinin (MAL II) was analysed, which preferentially recognizes α2,3 linked sialic acid (42). Both MDA-MB-468 and SK-BR-3 cells show MAL II binding, which was significantly reduced by more than 90% after treating the cells with neuraminidase (**Figures 1A, B**). EGFR and HER2 expression on tumor cell lines was analysed before and after treatment of the cells with neuraminidase from Vibrio cholerae (NEU-VC) using the EGFR and HER2 directed antibodies cetuximab and trastuzumab, respectively. Neither EGFR nor HER2 expression was altered by neuraminidase treatment (**Figure S1**). Similarly to NEU-VC, neuraminidase from Clostridium perfringens (NEU-CP) almost completely reduced MAL II binding on MDA-MB-468 and SK-BR-3 cells (**Figure 1C**). Reduction of sialic acid on tumor cells could also be achieved by using the sialyltransferase inhibitor (STi) P-3Fax-Neu5Ac (**Figures 1C, D**). In summary, the presence of α2,3 linked sialic acid on breast tumor cells could be significantly reduced with both neuraminidases and STi, while expression of the target antigens EGFR and HER2 was not affected, which is important for evaluating antibody-mediated effector functions such as ADCC.

### PMN express FcR and Siglecs, and Siglec binding to tumor cells is diminished after sialic acid reduction

**Figure 2:**
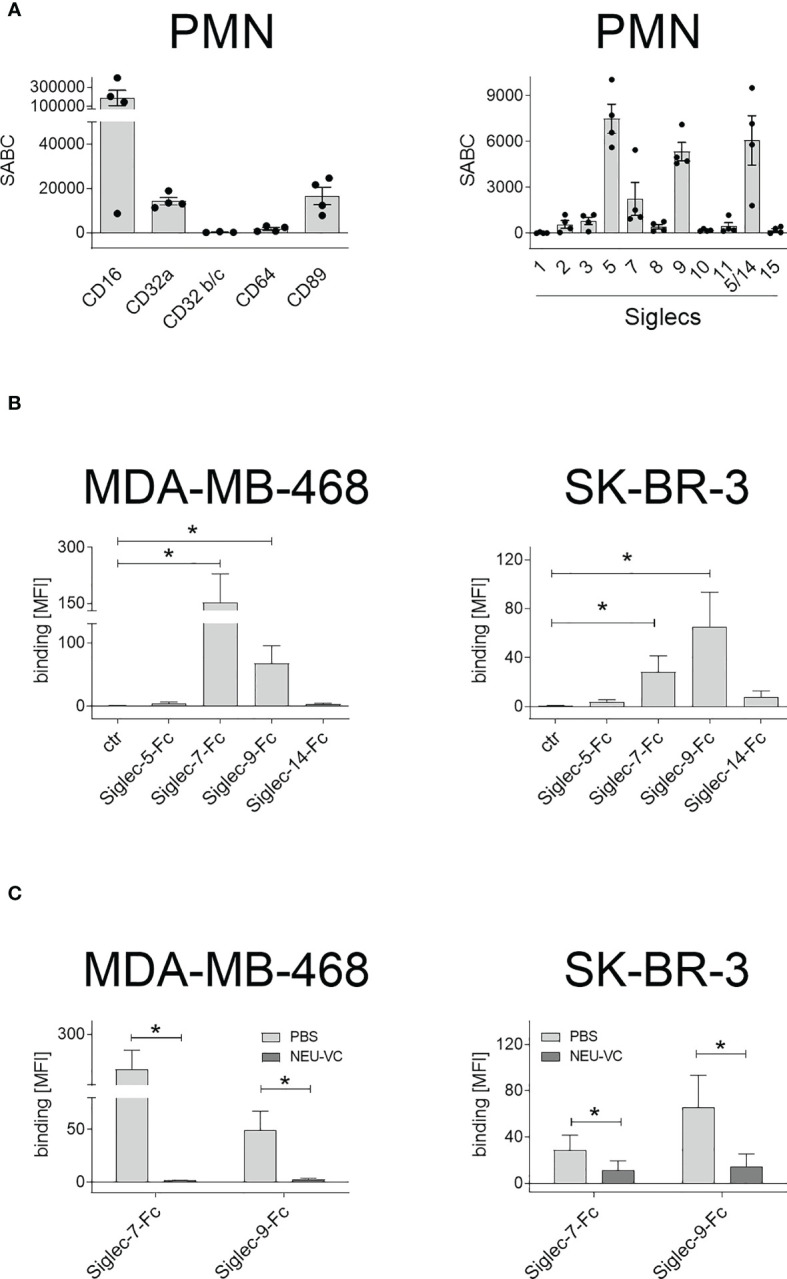
Expression of FcR and Siglecs on PMN, and soluble Siglec binding to tumor cells. **(A)** Expression of Fc receptors (FcR) (left panel) and Siglecs (right panel) on GM-CSF stimulated PMN stained with mouse monoclonal antibodies (5 µg/ml) and detected with FITC-conjugated goat anti-mouse Fcγ-specific F(ab)_2_ fragments. Antigen expression levels are depicted as mean specific antibody binding capacities (SABC) ± SEM of at least 3 different donors. **(B)** Siglec-binding epitopes on tumor cells were assessed by binding of soluble Siglec-Fc proteins (10 µg/ml) and detected with PE-conjugated goat anti-human Fcγ-specific F(ab)_2_ fragments. **(C)** Siglec-7-Fc and Siglec-9-Fc binding (10 µg/ml) on MDA-MB-468 and SK-BR-3 cells was reduced after treatment with neuraminidase (NEU-VC). The mean fluorescence intensity (MFI) values of more than three independent replicates are shown. * indicates significant differences (p < 0.05) compared to the controls (non-parametric one-tailed paired t-test).

Tumor cell killing by PMN in the presence of antibodies against TAA is mediated by Fc receptors (FcR). As such, expression of FcR on PMN was quantitatively analysed by flow cytometry (**Figure 2A**). As expected, human peripheral blood PMN from healthy donors were found to express high amounts of FcγRIII (CD16), lower amounts of FcγRIIa (CD32a) and FcαRI (CD89), while FcγRIIb/c receptors (CD32b/c) and the high affinity FcγRI receptor (CD64) were not expressed (**Figure 2A**, left panel). Next, expression of sialic acid-binding immunoglobulin-like lectins (Siglecs) on PMN was analysed (**Figure 2A**, right panel). PMN display a large panel of Siglecs, with highest expression for Siglec-5, Siglec-9 and Siglec-14. To assess if Siglec-5, Siglec-7, Siglec-9 and Siglec-14 on PMN may play a role in binding to sialic acid on tumor cells, soluble Siglec-Fc fusion proteins were analysed for their capacity to bind to MDA-MB-468 or SK-BR3. We included the expression of Siglec-7 ligands, because Siglec-7 has been shown to regulate other immune cells (43) and reported to be expressed by neutrophils as well (44). Both cell lines showed significant Siglec-7-Fc and Siglec-9-Fc binding, while binding of Siglec-5-Fc or Siglec-14-Fc was not detected or marginal (**Figure 2B**). The mean MFI values for Siglec-9-Fc were 71 ± 30 on MDA-MB-468 and 67 ± 28 on SK-BR-3 cells. Siglec-7-Fc binding on MDA-MB-468 cells had a mean MFI value of 151 ± 77, on SK-BR-3 the MFI value was much lower (**Figure 2B**). Importantly, treatment of MDA-MB-468 and SK-BR-3 cells with neuraminidases and STi (NEU-VC: **Figure 2C**; NEU-CP and STi: **Figure S2**) led to significantly reduced binding of Siglec-7-Fc and Siglec-9-Fc. These results show that human neutrophils express several Siglecs, some of which bind to tumor cells, and that Siglec-9 fulfils both requirements (expression on PMN and binding on tumor cells) to be a potential candidate in regulating antibody-dependent anti-tumor responses. Furthermore, treatment of tumor cells with neuraminidases and STi is a feasible approach to reduce soluble Siglec binding *in vitro*.

### Reducing sialic acid on tumor cells improves PMN mediated ADCC by therapeutic antibodies

**Figure 3:**
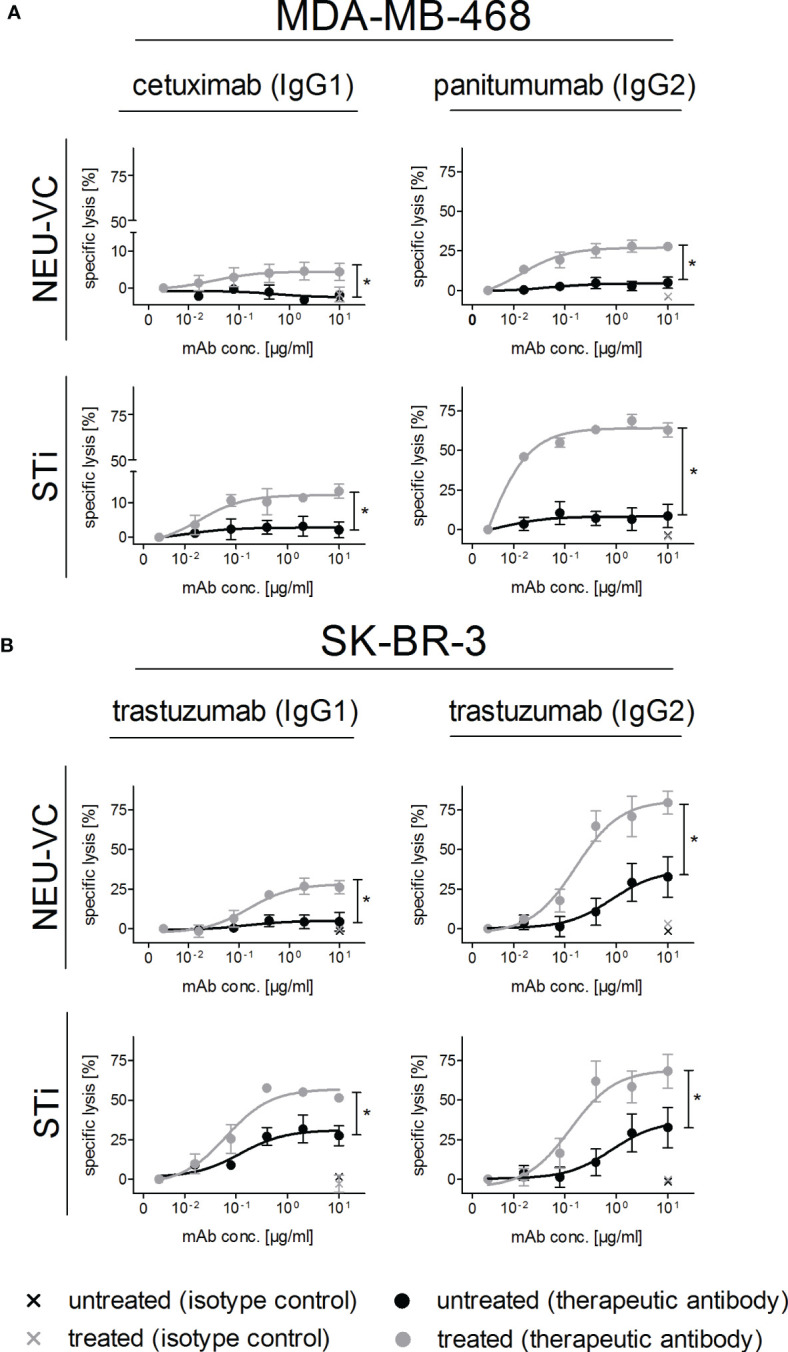
Reduced α2,3 linked sialic acid on tumor cells improves antibody-dependent PMN mediated ADCC. MDA-MB-468 **(A)** and SK-BR-3 **(B)** served as targets in [^51^Cr] release assays with GM-CSF (50 U/ml) stimulated PMN at an E:T cell ratio of 40:1. Tumor cells were treated (grey curves) with neuraminidase (NEU-VC) (0.1 U/ml) or with the sialyltransferase inhibitor (STi) (100 µM). IgG1 or IgG2 antibodies against EGFR or HER2 were used at indicated concentrations and control antibodies at 10 µg/ml. Shown are the mean values ± SEM as % specific lysis of at least 3 independent experiments with cells from different donors. Data were analysed by two-way ANOVA, and significant differences between treated and non-treated cells (*) are indicated.

Recent evidence suggests that upregulation of sialoglycans in malignant cells can reduce the tumor cell killing activity of myeloid effector cells (25, 26). To analyse the role of sialic acid in PMN mediated ADCC, neuraminidase or STi treated breast tumor cells were subjected to chromium release assays with the EGFR antibodies cetuximab (human IgG1) or panitumumab (human IgG2) for MDA-MB-468, and with the HER2 antibody trastuzumab as human IgG1 or IgG2 for SK-BR-3 - comparing the results to untreated cells. An IgG2 variant carrying the variable regions of trastuzumab was generated, and the purified protein was analyzed by SDS-PAGE (**Figure S3**). Binding to HER2 and FcRn at acidic pH was assessed by enzyme-linked immunosorbent assay (ELISA), whith no detectable FcRn binding at neutral pH. In general, PMN mediated lysis rates were significantly higher in tumor cells with reduced sialic acid compared to the untreated control cells, and the cytotoxic effects positively correlated with antibody concentrations (**Figure 3**). Panitumumab was more effective than cetuximab and besides the isotype, both antibodies also differ in their variable regions and bind to different, although overlapping epitopes (45). Maximal lysis rates were achieved with panitumumab in STi pretreated MDA-MB-468 cells with 64.0 ± 1.8% vs 8.0 ± 2.9% in untreated control cells (**Figure 3A**). To assess the impact of IgG1 vs IgG2 isotypes in more detail, respective isotype switch variants of trastuzumab were tested in ADCC against HER2 expressing SK-BR-3 cells. Here, the highest increment of SK-BR-3 lysis by PMN was achieved upon NEU-VC treatment with the IgG2 variant yielding 81.0 ± 6.2% lysis compared to 37.0 ± 9.8% lysis of untreated cells (**Figure 3B**). Lysis increments were also observed when sialic acids on tumor cells were cleaved by NEU-CP (**Figure S4**). Taken together, these results demonstrate that sialic acid reduction significantly enhanced PMN mediated cytotoxicity against breast tumor cells in the presence of tumor targeting IgG antibodies, with human IgG2 antibodies becoming particularly effective.

### Sialyltransferase inhibition improves EGFR-mediated tumor cell killing in a xenograft tumor model

**Figure 4:**
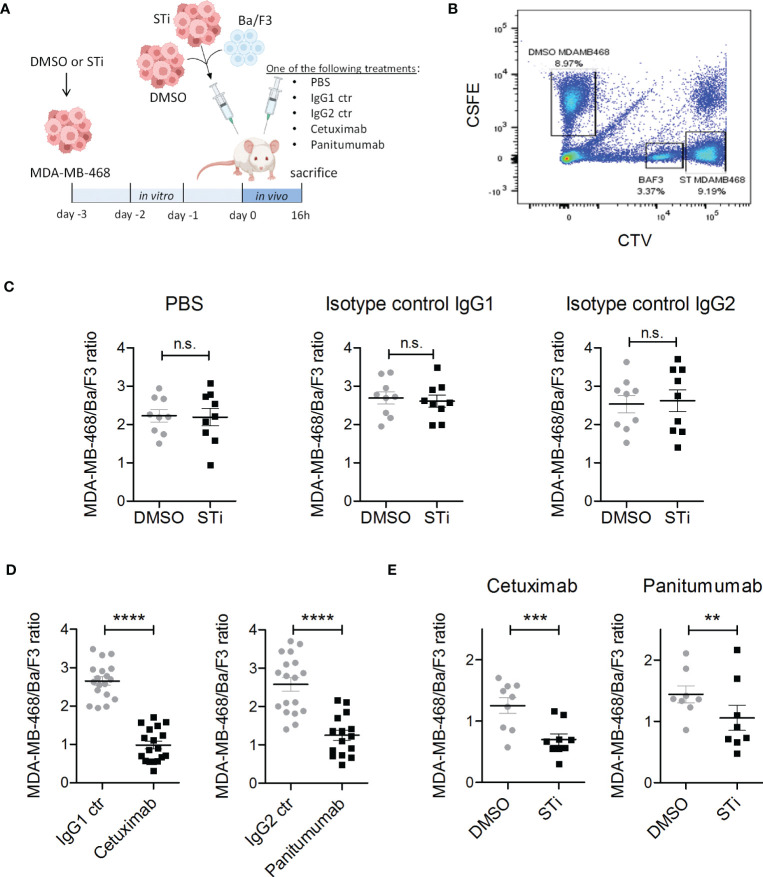
Sialyltransferase inhibition in tumor cells improves EGFR antibody therapeutic efficacy in a xenograft tumor model. **(A)** MDA-MB-468 tumor cells were treated for 3 days *in vitro* with 100 μM sialyltransferase inhibitor (STi) (MDA-MB-468 STi) or DMSO (MDA-MB-468 DMSO) prior to intraperitoneal (IP) injection into SCID mice. EGFR-negative murine Ba/F3 cells served as a recovery control. MDA-MB-468 DMSO, MDA-MB-468 STi and Ba/F3 cells were injected together at an initial ratio of 5:5:1. After injection, mice were separated into five groups receiving different treatments: PBS, cetuximab, panitumumab, IgG1 or IgG2 isotype controls (10 μg antibodies), respectively. After 16 h, mice were sacrificed and the numbers of residual tumor cells in the peritoneal fluid were evaluated by flow cytometry. **(B)** Gating strategy to discriminate MDA-MB-468 DMSO, MDA-MB-468 STi and Ba/F3 cells in flow cytometry. MDA-MB-468 DMSO cells were labelled with CSFE, MDA-MB-468 STi with CTV high and Ba/F3 with CTV low. **(C)** Effect of STi treatment on tumor cell recovery compared to DMSO in groups treated with PBS or isotype controls. **(D)** Reduction of MDA-MB-468 cells recovery from mice treated with EGFR antibodies with respect to their isotype. **(E)** Tumor cell recovery of STi treated cells compared to DMSO treated tumor cells in mice treated with EGFR antibodies. Results are presented as the mean ratio ± SEM of total MBA-MD-468 to Ba/F3 cells (at least 8 mice per group). Significant differences (*) were analysed by two-tailed, paired t-test (ns, not significant). **p < 0.01, ***p < 0.001, ****p < 0.0001. **Figure 4A** was created with BioRender.com.

Next, the effect of sialic acid removal on antibody therapy was investigated *in vivo* using xenografted SCID mice treated with human EGFR antibodies of different isotypes (**Figure 4A**). To avoid the expected *in vivo* toxicity of the STi inhibitor (46), MDA-MD-468 tumor cells were pre-treated with P-3Fax-Neu5Ac (100 μM) or DMSO *in vitro* for 3 days and together with Ba/F3 cells as recovery control injected into the peritoneal cavity of SCID mice as mixed population (MDA-MB-468 DMSO: MDA-MB-468 STi: Ba/F3) at a 5:5:1 ratio. After injection, mice were treated with either PBS, cetuximab (IgG1), panitumumab (IgG2), or isotype control antibodies (IgG1 or IgG2), each group containing at least eight mice. After 16 hours, mice were sacrificed and tumor load in the peritoneal cavity was analysed by flow cytometry (**Figures 4A, B**). To allow a clear distinction of the respective cell populations by flow cytometry after recovery from mice, DMSO treated tumor cells were labelled with CSFE fluorescent dye, STi treated tumor cells with CTV high and Ba/F3 cells with CTV low (**Figure 4B**). After ST inhibition *in vitro*, which does not interfere with tumor cell viability and proliferation (**Figure S5**), MAL II binding was performed to confirm the reduction of sialic acid prior to tumor cell injection in mice (**Figure S6A**). No significant differences were observed in the tumor cell number of STi treated cells compared to DMSO cells in the groups of mice treated with PBS or isotype control antibodies, which indicates that the absence of sialic acid alone is not sufficient to improve tumor cell clearance (**Figure 4C**). On the other hand, both EGFR antibodies significantly reduced the overall MDA-MB-468 tumor cell number compared to control mice treated with the respective isotype control antibody (**Figure 4D**). Importantly, the tumor cell number of STi treated cells was significantly decreased compared to DMSO control cells in both mouse groups treated with either cetuximab or panitumumab (**Figure 4E**). Interestingly, STi treated tumor cells had lower levels of sialic acid molecules on their cell surface compared to the DMSO treated cells, even after 16h intraperitoneal incubation, which excludes sialic acid recovery *in vivo* after previous ST inhibition *in vitro* (**Figure S6B**). Together, our results demonstrate that the reduction of sialic acid on tumor cells, obtained by sialyltransferase inhibition, enhances the *in vivo* anti-tumor activity of EGFR-targeting antibodies.

### Blocking Siglec-9 on PMN improves ADCC by therapeutic IgG antibodies

**Figure 5:**
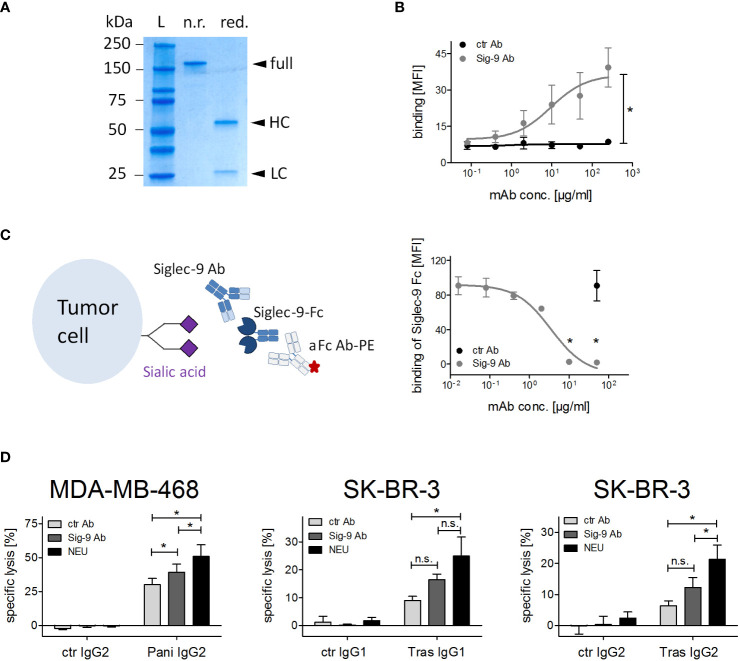
Siglec-9 blockade on PMN improves ADCC by therapeutic IgG antibodies. **(A)** SDS-PAGE under non-reducing (n.r.) and reducing (red.) conditions of the Siglec-9 antibody. HC, heavy chain; LC, light chain; L, protein ladder. **(B)** Binding of Siglec-9 antibody on PMN. A non-binding IgG2σ antibody served as control, anti-human kappa-FITC was used for detection. Mean of the mean fluorescence intensity (MFI) values ± SEM of 3 independent experiments with PMN from 3 different donors are shown. Data were analysed by two-way ANOVA, and * indicates a significant difference. **(C)** Binding of Siglec-9-Fc fusion protein (5 µg/ml) on tumor cells is blocked with increasing concentrations of the Siglec-9 antibody. The non-binding IgG2σ antibody was used as control (50 μg/ml). The Siglec-9-Fc fusion protein was detected by a goat anti-human IgG PE-conjugated antibody. Mean MFI values ± SEM of 3 independent experiments are shown. Data were analysed by t-student test and significant blocking is depicted by *. **(D)** PMN ADCC with MDA-MB-468 or SK-BR-3 was performed in the presence of panitumumab IgG2 (10 µg/ml), trastuzumab (10 µg/ml), or trastuzumab IgG2 (2 µg/ml) using the Siglec-9 blocking antibody (20 µg/ml). The effect of the Siglec-9 blocking antibody was also compared to neuraminidase treatment (0.1 U/ml). At least 3 independent experiments with different donors, are displayed as mean values ± SEM (% specific lysis). Data were analysed by two-way ANOVA with Bonferroni post-test correction. Significant differences (*) are indicated. n.s., not significant.

Removal of sialic acid from tumor cells potentially affects interactions with different Siglecs on effector cells. To directly analyse the impact of Siglec-9 for PMN mediated ADCC, a Siglec-9 blocking antibody was produced from published sequences (24), employing an immune effector function silent Fc region (IgG2σ) (41). To confirm the purity of the Siglec-9 IgG2σ, SDS-PAGE followed by Coomassie blue staining was performed under non-reducing and reducing conditions (**Figure 5A**). The Siglec-9 antibody showed dose-dependent binding on PMN isolated from peripheral blood of healthy human donors as assessed by flow cytometry (**Figure 5B**). Next, the blocking capacity of the Siglec-9 antibody was evaluated by measuring its ability to exclude binding of the Siglec-9-Fc fusion protein to tumor cells (**Figure 5C**, left panel). Binding of the Siglec-9-Fc fusion protein to tumor cells was dose dependently blocked by the Siglec-9 antibody, but not by an isotype control IgG2σ Fc antibody (**Figure 5C**, right panel). At concentrations of 10 µg/ml and higher, the Siglec-9 antibody was able to completely block Siglec-9-Fc fusion protein binding to tumor cells. In ADCC assays (**Figures 5D**), the lysis rates of MDA-MB-468 with panitumumab were significantly increased from 29.7 ± 6.0% to 43.2 ± 6.1% in combination with the Siglec-9 blockade (**Figure 5D**). The lysis rates of SK-BR-3 with trastuzumab IgG1 were increased from 8.9 ± 1.5% to 16.5 ± 1.9% in the presence of the Siglec-9 antibody (**Figure 5D**). Similar results as with trastuzumab were observed with the IgG2 isotype version of trastuzumab (**Figure 5D**). The effect of the Siglec-9 blocking antibody, however, tended to be less pronounced than with sialic acid depletion of tumor cells by neuraminidase treatment (specific lysis: 56.7 ± 8.8% for MDA-MB-468; 25.0 ± 6.8% for SK-BR-3 with trastuzumab IgG1 and 21,3 ± 4,6% with trastuzumab IgG2) (**Figure 5D**). This finding may indicate that Siglec-9 is not the only regulator of ADCC activity by PMN.

### Siglec-9 binds to tumor associated carbohydrate antigens

**Figure 6:**
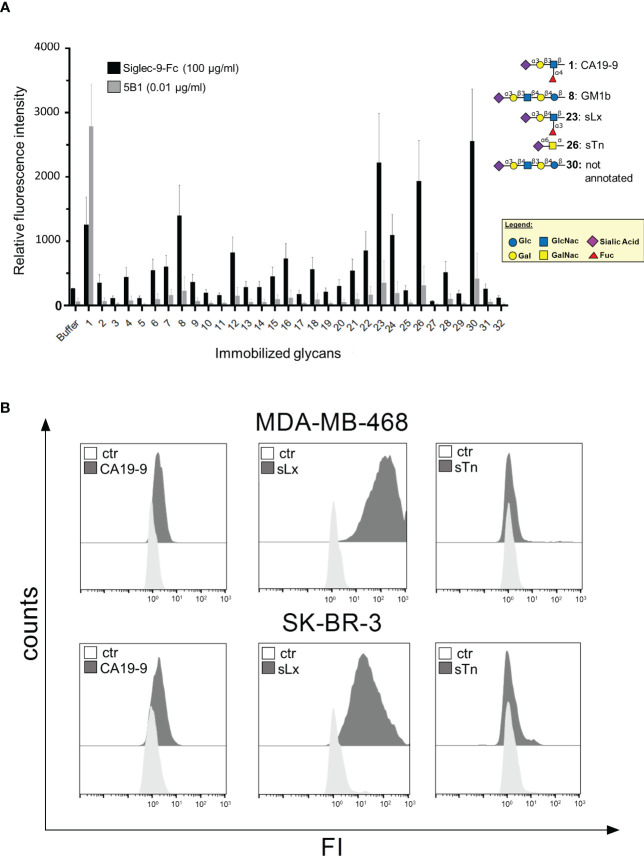
Siglec-9-Fc binds to tumor associated carbohydrate antigens. **(A)** Tumor associated carbohydrate antigens (TACAs), with O-glycosylation structure bound to an amino-pentanol linker, were analysed in a glycan array by their ability to bind soluble Siglec-9-Fc (100 µg/ml). Reference antibody 5B1 (0,01 µg/ml) binds only to CA19-9. Shown are mean values +/- SEM of the 3 individual experiments. In each experiment, six data points for each sugar were collected, up to 2 were excluded if the background was too high. **(B)** Expression of CA19-9, sLewisX (sLx) and sTn on MDA-MB-468 and SK-BR-3 cells as analysed by indirect flow cytometry. Binding of primary antibodies (mIgG1 or mIgM, 10 μg/ml) (grey) were detected with FITC-conjugated goat anti-mouse IgG and IgM-specific F(ab)_2_ fragments. Representative histograms of 3 replicates are shown.

With the intention to identify the ligand for Siglec-9 on our tested breast cancer cell lines we employed a broad panel of chemically synthesized TACAs (**Figure S7** and **Table S1**). Binding of soluble Siglec-9-Fc to glycan structures was analysed in an immunofluorescence based assay. Out of 32 tested glycans, Siglec-9-Fc was found to predominantly bind to TACAs terminating with N-acetylneuraminic acid (Neu5Ac): sialyl-Lewis-A (sLewisA/CA19-9, #1), sialyl-Thomsen-nouvelle (sTn, #26) and sialyl-Lewis-X (sLewisX, #23) (**Figure 6A**). Immunofluorescence analyses with specific mouse antibodies against CA19-9, sLewisX and sTn demonstrated that MDA-MB-468 and SK-BR-3 cells express low levels of CA19-9, high levels of sLewisX and do not express sTn (**Figure 6B**). Together, these results suggest that sLewisX or CA19-9 could be putative ligands for Siglec-9, but their role in regulating PMN mediated cytotoxicity requires further studies.

## Discussion

In this study, we analysed the impact of sialic acid/Siglec interactions on modulating the activity of neutrophils (PMN) in antibody mediated tumor cell killing. In the tumor microenvironment, neutrophil-like cells constitute a significant proportion of the cellular infiltrate and are often described as myeloid-derived suppressor cells (MDSC) (47). However, neutrophil heterogeneity has been described to discriminate between pro- and anti-tumorigenic N2 and N1 neutrophils, respectively (29, 33). In peripheral blood, PMN are the most abundant leukocyte population and constitute our first life of defence against bacteria and fungi. They can be rapidly recruited to inflamed sites and also have high cytotoxic capacity in tumor cell killing. Thus, recruiting this cell population for antibody-based immunotherapy may be a promising strategy to enhance anti-tumor immunity (29, 48).

Except for the interaction of CD47 on tumor cells with SIRPα on neutrophils (49), the knowledge about immune checkpoint molecules on neutrophils is limited (31). In this study, we evaluated the sialic acid/Siglec-9 axis as a potential myeloid checkpoint in antibody dependent tumor cell killing by neutrophils using breast cancer cell lines as model.

Sialic acid with an α2,3 glycosidic linkage was detected on MDA-MB-468 and SK-BR-3 breast tumor cells, as has been previously reported for these and other breast tumor cell lines (50). In many aggressive tumors, sialic acid metabolism is upregulated and different sialyltransferases are highly expressed, being associated with increased metastatic potential (51). Indeed, upregulation of surface sialic acid on tumor cells affects the activation of immune cells e.g. via Siglec receptors, mostly leading to dampened immune responses and tumor escape (19, 20, 52–54). While this has been extensively described for NK cells, T cells and macrophages, the knowledge about the role of sialic acid/Siglec interactions for the regulation of neutrophil activity in the context of tumor biology is limited, especially when engaged by therapeutic antibodies (16, 21, 28, 55–57).

Here, we analysed the expression of Siglecs on human neutrophils isolated from peripheral blood. Neutrophils are shown to predominantly express Siglec-5, -9, and -14 and to lower extent other Siglecs like e.g. Siglec-7, as previously described (58, 59). The expression of Siglec-14 on PMN, however, remains to be shown, since all available antibodies against Siglec-14 may cross-react with Siglec-5 due to the sequence identity between these paired Siglec receptors that balance immune responses to pathogens (60) and other models of breast tumor cells (61) than the ones used in our study. Since the Siglec-13 gene is deleted in humans, and Siglec-4, -6, and -12 are exclusively expressed on neural cells, placental trophoblasts or epithelial cells (12), respectively, these Siglecs were not analysed in our study. Next, we wanted to determine whether human Siglec receptors can bind cell surface sialic acid on human breast tumor cell lines. Our flow cytometric studies with soluble Siglec-Fc reagents show that Siglec-7 and Siglec-9 were able to bind to MDA-MB-468 and SK-BR-3 cells, which makes them suitable targets for both NK cells and PMN. In line with our findings, histological sections of human carcinomas revealed up-regulation of Siglec-9 ligands on breast tumor cells and Siglec-9 positive infiltrating immune cells (54). The overexpression of Siglec-7 and Siglec-9 ligands detected in different histological types of tumors and the predominant expression of Siglec-7 on NK cells and Siglec-9 on neutrophils may suggest that these Siglecs are the prominent regulators of innate immune cells (19).

The levels of α2,3 linked sialic acid and Siglec ligands could be reduced by treatment of tumor cells with two neuraminidases or a sialyltransferase inhibitor. We show that removal of α2,3 linked sialic acids resulted in enhanced neutrophil cytotoxicity *in vitro* in the presence of human IgG1 and IgG2 antibodies against HER2 and EGFR positive breast tumor cells. Similar results were also observed with human IgA2 antibodies (62). Interestingly, human IgG2 antibodies particularly benefited from this intervention. Human IgG2 has higher binding affinity for the ITAM-containing FcγRIIa (CD32a) receptor compared to the GPI-linked FcγRIIIb (CD16) receptor, which explains its higher activity in PMN mediated ADCC compared to human IgG1 (35, 63, 64). However, additional studies are required to explore why human IgG2 antibodies particularly benefit from interference with sialic acid/Siglec interactions. In contrast to Läubli et al. (54), the increase in neutrophil cytotoxicity was only seen in our study in the presence of tumor targeting antibodies, which is relevant for tumor directed antibody therapy. Importantly, sialyltransferase inhibition combined with EGFR antibodies was also effective in reducing the tumor load in a xenografted *in vivo* model. Remarkably, bacterial pathogens evade neutrophil immune responses by mimicking host cell sialylation on their capsular polysaccharides that activate Siglec-9 (65, 66). Tumor cells may also adopt this strategy to evade the immune response via Siglec-9 by upregulating self-associated molecular patterns (SAMPs) such as sialic acid. Our results indicate that Siglec-9 is a key regulator of PMN activity, because it is one of the predominantly expressed Siglecs on human neutrophils and its blockade enhanced PMN ADCC.

Next, we attempted to identify the ligand of Siglec-9 responsible for PMN mediated cytotoxicity against tumor cells. In a fluorescence-based assay using chemically engineered glycans, sLewisA, sLewisX and sTn were identified as the most prominent glycans that bind Siglec-9. Binding of Siglec-9 to sLewisX has been previously described (67) and recently, sLewisA and sLewisX have been reported as MYC-driven ligands of Siglecs (68). The breast tumor cell lines used in our study express low levels of sLewisA/CA19-9 and high levels of sLewisX on the cell surface. However, they do not express sTn, another glycan structure that binds to Siglec-9. Taken together, the predominant ligand for Siglec-9 in the tested breast tumor cell lines still needs to be identified; however sLewisA and especially sLewisX may be putative candidates. Despite of preferential binding of Siglec-9 to N-glycans (69), we could show in our glycan array that Siglec-9 also binds to sialylated TACAs with typical O-glycosylated structures. In this regard, it is important to mention that also the O-linked sialylated glycan MUC1-ST has been reported to be a key player in the regulation of pro-tumor activity of myeloid cells via Siglec-9 (52). Besides that, our Siglec-9 blocking antibody was not as efficient as neuraminidase treatment in enhancing PMN ADCC, which suggests that Siglec-9 may not be the only reason why sialic acid reduction enhances PMN activation. For example, a recent study demonstrated that also Siglec-7 on PMN can contribute to their ADCC activity (70), although it is only expressed at low levels. Additionally, a sialic acid rich glycocalyx generates negative charges on the cell surface which modifies cell-cell interactions, providing a steric hindrance and an electrostatic repulsion of effector cells. This mechanism has been shown, for example, to impede tumor cell phagocytosis by macrophages (71, 72). Further supporting this hypothesis, enhanced tumor cell killing by cytotoxic CD8+ T cells caused by sialic acid reduction, was explained by increased clustering of T cells with tumor cells (16). Furthermore, the addition of terminal sialic acid to the glycocalyx may mask underlying galactoses, which can modulate immune responses through galectins (73) and function as “eat me signal” e.g. on apoptotic lymphocytes (74). A similar mechanism has been reported for desialylated erythrocytes and lymphocytes that expose galactose leading to the sequestration from circulation by galectin expressed on liver and spleen macrophages (75).

An important limitation in studies of the sialic acid/Siglec axis is the difference of Siglecs between humans and mice, which e.g. impeded us to test our Siglec-9 blocking antibody *in vivo*. While humans have nine CD33-related Siglecs, mice only have five, making it difficult to match Siglec orthologues between species (13). Additionally, a different nomenclature is employed: Siglecs are numbered in humans and lettered in mice. Within the mouse Siglecs containing an ITIM as intracellular signalling motif, Siglec-F is found mainly on eosinophils and Siglec-G on B cells, and these are the closest functional orthologues of Siglecs-8 and -10 in humans, respectively. Siglec-E is expressed by neutrophils, monocytes and dendritic cells and is the orthologue of Siglec-9 (12). To address the problem of different Siglecs in mice and humans Ravetch and colleagues generated an immunocompetent transgenic mouse expressing Siglec-7 and -9, showing the therapeutic potential of blocking antibodies against these Siglecs for tumor immunotherapy (25).

Additional approaches are pursued to exploit the knowledge about the impact of sialic acid/Siglec interactions in tumor immunotherapy. Unfortunately, systemic applications of neuraminidases or sialyltransferase inhibitors - used here to enhance PMN ADCC *in vitro* and for pre-treatment of tumor cells before *in vivo* injection – are probably so far not feasible for *in vivo* application due to their potential toxicity. To overcome the problem of systemic toxicity, a HER2 antibody has been conjugated to sialidase to target sialidase activity specifically to the tumor site (53). *In vivo*, this HER2 antibody-sialidase conjugate improved the antitumor immune response in mice through the engagement of Siglec-E^+^, CD11b^+^ tumor-infiltrating myeloid cells (26). A novel approach to target sialic acid on tumor cells is to combine tumor targeting antibodies with engineered Siglecs in bispecific molecules called AbLecs, which were demonstrated to be more effective than combinations of the respective antibodies and soluble Siglecs (70).

Our findings provide evidence that Siglec-9 on PMN plays a critical role in inhibiting neutrophil mediated tumor cell killing by therapeutic antibodies. Reducing hypersialylation of tumor cells consistently increased the efficacy of HER2 or EGFR antibodies. In conclusion, the sialic acid/Siglec-9 axis is an immune checkpoint for PMN, besides NK cells, T cells and macrophages, and disrupting this interaction may be a strategy to enhance monoclonal antibody efficacy.

## Data availability statement

The original contributions presented in the study are included in the article/**Supplementary Material**. Further inquiries can be directed to the corresponding author.

## Ethics statement

The studies involving human participants were reviewed and approved by Ethics Committee of the Christian-Albrechts-University of Kiel. The patients/participants provided their written informed consent to participate in this study. The animal study was reviewed and approved by Animal Ethical Committee of the UMC Utrecht.

## Author contributions

ML, CC, MB, MK, CG, NB, SM, SF performed the experiments and data analysis. ML, RB, and TV designed the *in vitro* experiments and wrote the manuscript. CC, JJ and JL designed and performed the *in vivo* experiments. TV supervised the project. LB, JA, PS, MP, and all other authors critically revised and reviewed the manuscript and approved its submission. All authors contributed to the article and approved the submitted version.
