# Supplementary material for: Disruption of the sialic acid/Siglec-9 axis improves antibody-mediated neutrophil cytotoxicity towards tumor cells
Source: Front Immunol. 2023 Jun 6;14:1178817. doi: 10.3389/fimmu.2023.1178817 (PMC10279866; doi:10.3389/fimmu.2023.1178817)
Supplement: Supplementary file 1 [file DataSheet_1.docx]

Disruption of the sialic acid/Siglec-9 axis improves antibody-mediated neutrophil cytotoxicity towards tumor cells

Marta Lustig^1^, Chilam Chan^2^, J. H. Marco Jansen^2^, Maria Bräutigam^3^, Max A. Kölling^1^, Carina Lynn Gehlert^4^, Niklas Baumann^1^, Simone Mester ^5,6^, Stian Foss ^5,6^, Jan Terje Andersen^5,6^, Lorenz Bastian^7^, Peter Sondermann^3^, Matthias Peipp^4^, Renate Burger^1^, Jeanette H.W. Leusen^2^, Thomas Valerius^1#^

^1^ Division of Stem Cell Transplantation and Immunotherapy, Department of Medicine II, Christian-Albrechts-University Kiel and University Medical Center Schleswig-Holstein Campus Kiel, Kiel, Germany

^2^ Center for Translational Immunology, University Medical Center Utrecht, Utrecht, The Netherlands

^3^ Tacalyx GmbH, Berlin, Germany

^4^ Division of Antibody-Based Immunotherapy, Department of Medicine II, Christian-Albrechts-University Kiel and University Medical Center Schleswig-Holstein Campus Kiel, Kiel, Germany

^5^ Institute for Clinical Medicine, Department of Pharmacology, University of Oslo and Oslo University Hospital, Oslo, Norway

^6^ Institute for Clinical Medicine, Department of Immunology, University of Oslo and Oslo University Hospital, Oslo, Norway.

^7^ Department of Medicine II, Christian-Albrechts-University Kiel and University, Medical Center Schleswig-Holstein, Kiel, Germany

*** Correspondence:**Thomas Valerius, MD

[t.valerius@med2.uni-kiel.de](mailto:t.valerius@med2.uni-kiel.de)

# Supplementary Data

## Flow cytometry

Immunofluorescence acquisition was performed using flow cytometry (Navios, Beckman Coulter). Cetuximab and trastuzumab (10 µg/ml, 30 min, 4 °C) were used to detect EGFR and HER2 receptors on tumor cells. Nonbinding IgG1 was used as control antibody. Binding was detected using phycoerythrin (PE)-conjugated goat anti-human Fcγ-specific F(ab)2 fragments (30 min, 4 °C) (Jackson ImmunoResearch Laboratories, West Grove, PA, USA). For detection of α2,3-linked sialic acids, cells were incubated with biotinylated Maackia amurensis leukagglutinin II (MAL II) (Vector Laboratories) (5 µg/ml, 20 min, 4 °C) and with streptavidin-PE (1:50, BioLegend). Antigen expression levels of Siglecs and Fc receptors on PMN were quantified by determination of specific antigen binding capacities (SABC) of monoclonal mouse antibodies (Biolegend) (50 µg/ml, 1 h, 4 °C) using the QIFI KIT (Agilent DAKO). Antibodies against human Siglec-10 and Siglec-14 were purchased from ThermoFisher. Binding of mouse antibodies was detected using FITC-conjugated goat anti-mouse Fcγ-specific F(ab)2 fragments (30 min, 4 °C) (Jackson ImmunoResearch Laboratories). Recombinant soluble human Siglec-Fc chimera proteins (R&D Systems) were used to assess the presence of Siglec-binding epitopes on tumor cells (10 µg/ml for 1 h at 4 °C). Binding was detected using PE-conjugated goat anti-human Fcγ-specific F(ab)2 fragments (30 min, 4 °C). Binding of the Siglec-9 blocking antibody was assessed on PMN and an irrelevant human IgG2σ Fc antibody (250 μg/ml) was used as control antibody. Binding was detected by a mouse anti-human kappa light chain FITC-conjugated antibody (30 min, 4 °C) (Southern Biotech). Anti-human CA19-9 mIgG1, sTn mIgG1 and sLewisX IgM were used to detected glycan

## Antigen binding

96-well ELISA plates (Costar) were coated with 1.0 μg/ml recombinant human Her2 (Sino Biological) diluted in PBS (Merck), and incubated overnight at 4°C. The next day, plates were blocked for 1 hour at room temperature (RT) w/shaking, using PBS with 4% skimmed milk (S) (ITW reagents). The plates were washed 4 times with PBS containing 0.05% Tween 20 (T) (Merck) before 100 μl of titrated amounts of purified trastuzumab IgG2 in PBS/S/T were added, and the plates incubated for 1 hour at RT w/shaker. The plates were washed again, and 100 μl of an alkaline phosphatase (ALP) conjugated anti-human kappa LC (Southern Biotech) or anti-human IgG-Fc (Merck) were added (1:2000) and incubated for 1 hour at RT w/shaking. After the last wash, bound proteins were visualized by addition of 100 μl of ALP substrate (1 mg/ml phosphate in diethanolamine buffer) (Merck). Absorbance was measured at 405 nm with a Sunrise spectrophotometer (TECAN).

## Human FcRn binding

96-well ELISA plates (Costar) were coated and blocked as described above, followed by addition of titrated amounts of trastuzumab variants and incubated for 1h art RT w/shaking. Then, the plates were washed with either PBS/T pH 5.5/pH 7.4, and 100 μl 2 μg/ml of human FcRn-GST in PBS/S/T pH 5.5/pH 7.4 was added. After 1 hour of incubation at RT w/shaking, plates were washed again with pH 5.5/pH 7.4, and 100 μl horseradish peroxidase (HRP) conjugated goat anti-GST antibody (Rockland), diluted (1:8000) in PBS/S/T with pH 5.5/7.4 was added to the plates, following 1 hour of incubation at RT w/shaking. Plates were then washed again, and 100 μl of 3,3',5,5'-tetramethybenzidine (TMB) substrate solution (Merck Millipore) was added to the wells. Absorbance was measured at 620 nm with a Sunrise spectrophotometer (TECAN), and the reaction was stopped by adding 50 μl 1 M HCl, and then measured again at 450 nm.

## ADCC assay

ADCC was analysed by [^51^Cr] release assay as previously described [^38^](#_ENREF_38). Tumor cells were labelled with radioactive [^51^Cr]-chromium as sodium chromate (Hartmann Analytic) for 2 h, washed and then added together with antibodies and GM-CSF (50 U/ml, CellGenix GmbH) stimulated PMN at an effector-to-target cell (E:T) ratio of 40:1 (200.000 : 5.000). After incubation for 3 h at 37 °C, the supernatants from triplicate cultures were collected, added to Optiphase HiSafe 3, and measured in a MicroBeta TriLux Scintillation & Luminescence Counter (Perkin Elmer). ^51^Cr-release from triplicates was measured in counts per minute (cpm). Specific lysis was calculated by following formula:

$$percentage of specific lysis=\frac{experimental cpm-basal cpm}{maximal cpm- basal cpm}\times100$$

Maximal release of [^51^Cr] by tumor cells was achieved by adding Triton-X-100 (2 % v/v), and the basal release was measured in the absence of antibodies and presence of effector cells.

## Production and purification of a Siglec-9 blocking antibody

The human Siglec-9 antibody, carrying a modified Fc domain to prevent FcγR binding (IgG2σ: amino acid substitutions V234A/G237A/P238S/H268A/V309L/A330S/P331S) [^39^](#_ENREF_39), was generated from de novo synthesized variable light (VL) and heavy (VH) chains according to published sequences (clone: mAbA) [^21^](#_ENREF_21). VL and VH sequences were ligated into the expression vectors pSEC-LC-kappa and pSec-HC-IgG2σ, encoding either the human antibody κ light chain or the IgG2σ heavy chain, respectively. Plasmid DNA was purified endotoxin-free by Nucleo Bond 2000 EF (Macherey-Nagel). CHO-S cells were transiently co-transfected with the plasmids encoding the heavy and light chain by electroporation using the MaxCyte STX large scale electroporation system (MaxCyte). The antibody was purified from the supernatant by Capture Select IgG-CH1-XL affinity matrix (ThermoFisher) following the manufacturer’s recommendations.

# Supplementary Figures and Tables

## Supplementary Figures

### Supplementary Figure 1


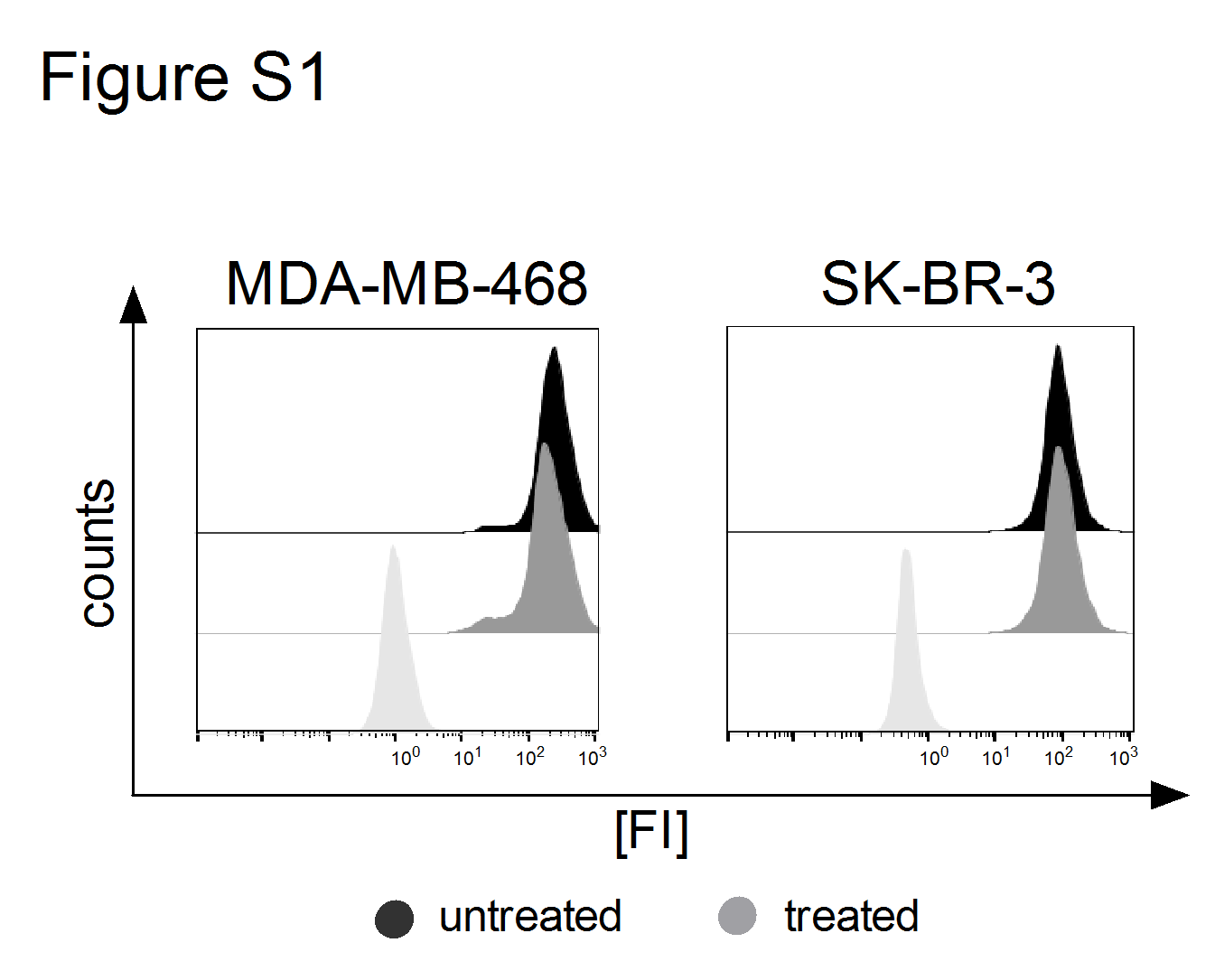


**Figure S1: NEU-VC treatment does not affect binding of therapeutic antibodies to EGFR and HER2 on tumor cells.** EGFR and HER2 expression on MDA-MB-468 and SK-BR-3 cells, treated with neuraminidase of Vibrio cholerae (NEU-VC) (0.1 U/ml) (grey) or untreated (black). Cells were stained with cetuximab (left panel), trastuzumab (right panel), or IgG1 isotype control (both panels; all 10 µg/ml). PE-conjugated goat anti-human Fcγ-specific F(ab)_2_ fragments were used for detection. Shown are representative histograms.

### Supplementary Figure 2


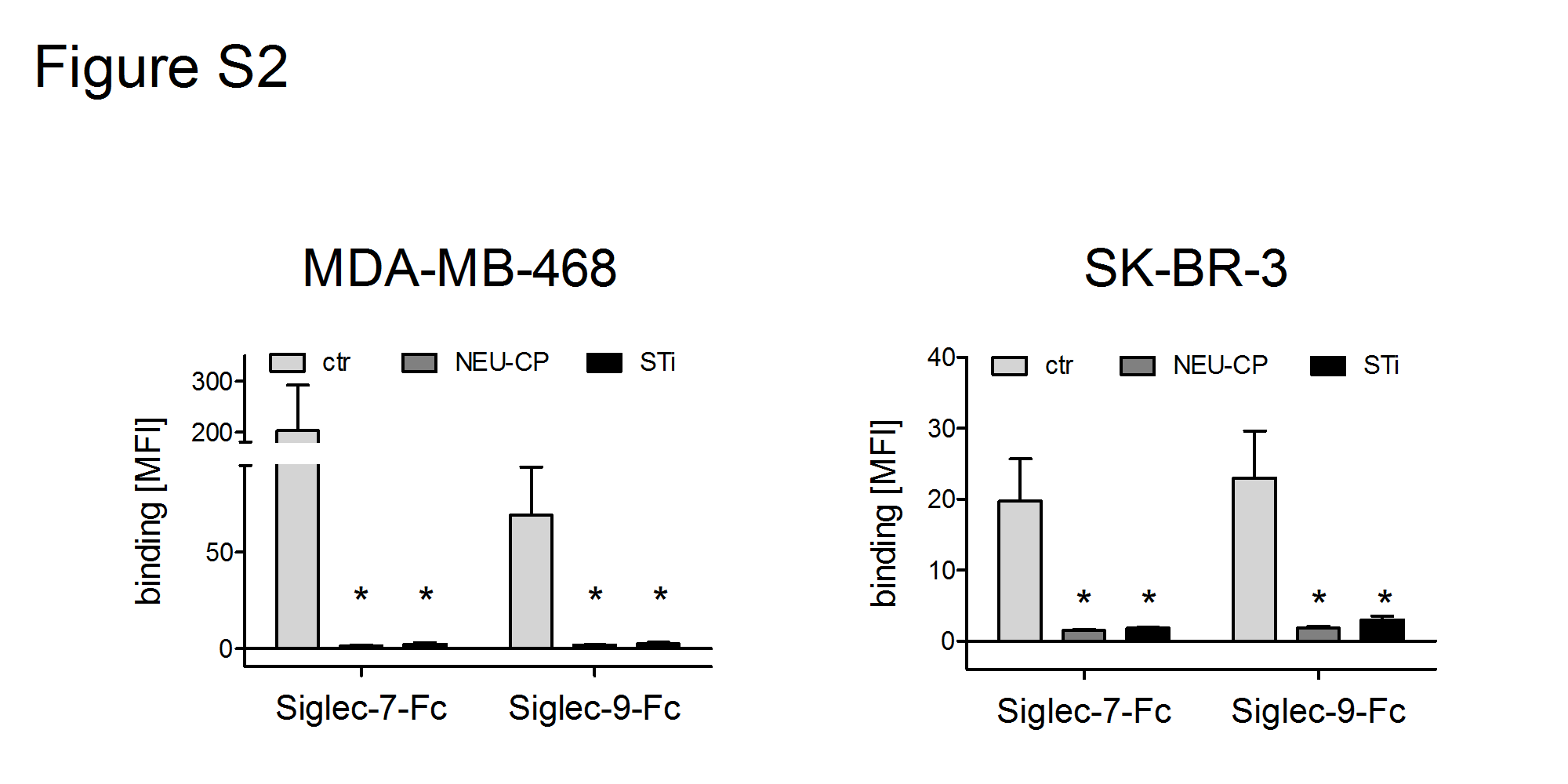


**Figure S2: Siglec-7 and Siglec-9 ligands are reduced by NEU-CP and STi treatment.** Siglec-7-Fc and Siglec-9-Fc binding (10 µg/ml) on MDA-MB-468 and SK-BR-3 cells was reduced after treatment with neuraminidase (NEU-CP; 1 U/ml) or with the sialyltransferase inhibitor (STi; 100µM). The mean fluorescence intensity (MFI) values of more than three independent replicates are shown. * indicates significant differences (p < 0.05) compared to the untreated cells (ctr) (parametric two-way ANOVA with Bonferroni post-test analysis).

### Supplementary Figure 3


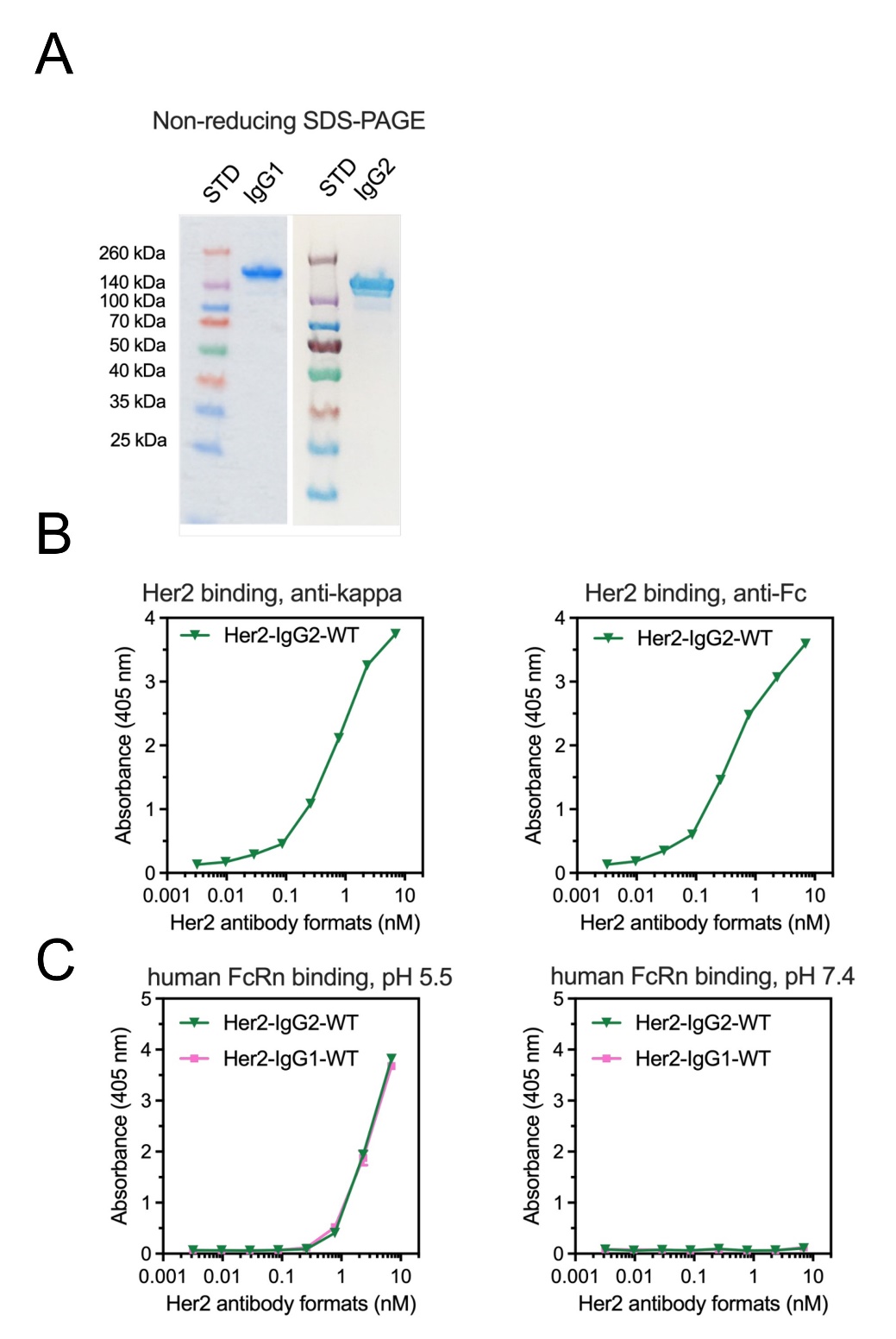


**Figure S3: Trastuzumab IgG2 production.** (**A**) Non-reducing SDS-Page gel of trastuzumab IgG1 and IgG2 variants. (**B**) HER2 binding ELISA showing binding of titrated amounts of trastuzumab IgG2 to recombinant HER2 coated in wells. Shown as mean ± SD of duplicates. (**C**) Human FcRn ELISA results showing receptor binding of titrated amounts of trastuzumab IgG1 and IgG2 variants at pH 5.5 and 7.4. Shown as mean±SD of duplicates.

### Supplementary Figure 4


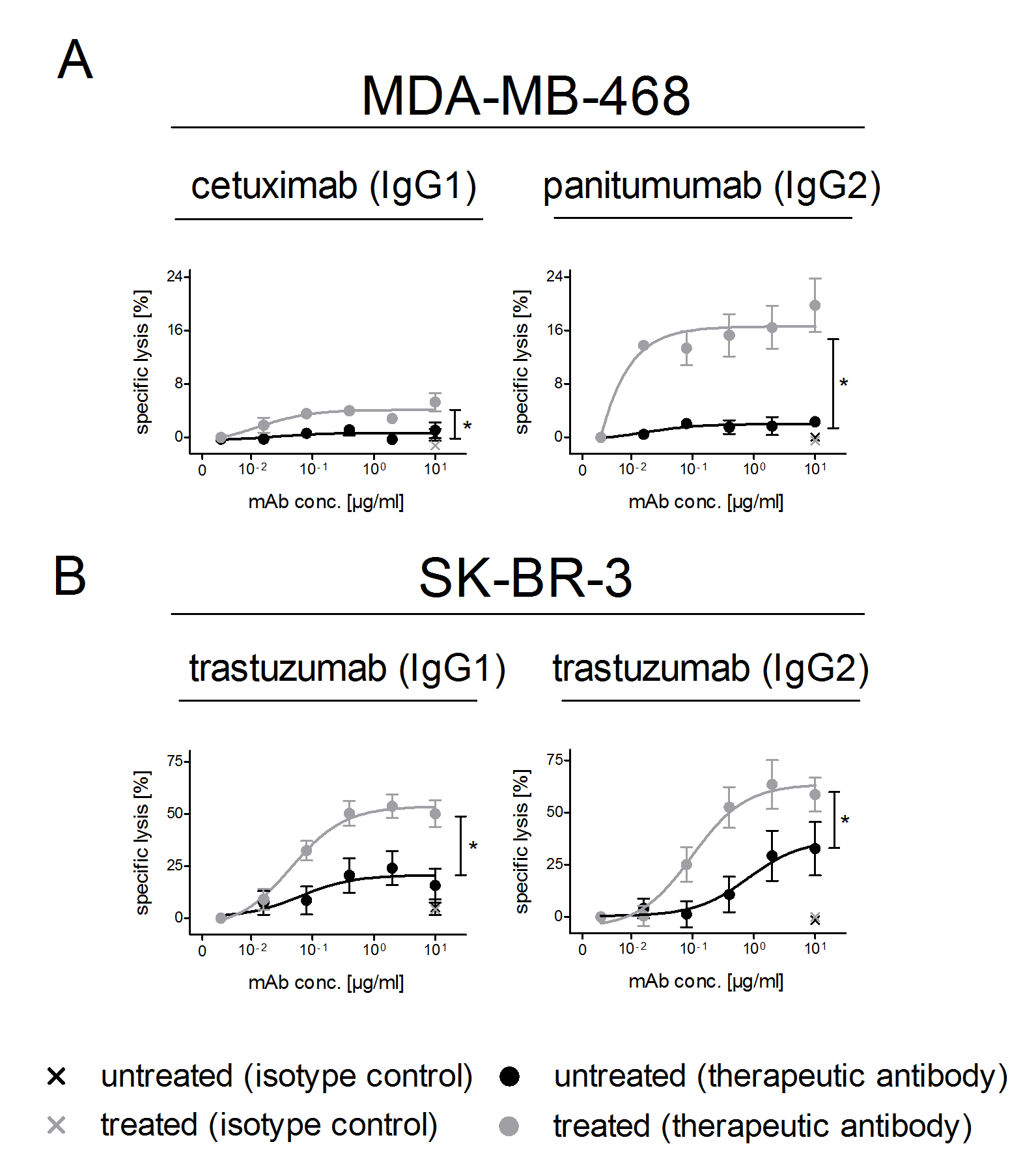


**Figure S4: NEU-CP treatment of tumor cells enhances PMN mediated ADCC by therapeutic antibodies**. The cell lines MDA-MB-468 and SK-BR-3 were used as targets in [^51^Cr] release assays with GM-CSF (50 U/ml) stimulated PMN at an E:T cell ratio of 40:1. Tumor cells treated with NEU-CP neuraminidase (1 U/ml) are depicted by grey curves and PBS treated control cells by black curves. EGFR or HER2 specific IgG1 or IgG2 antibodies were used at the indicated concentrations, isotype control antibodies were used at 10 µg/ml. Shown are the mean values ± SEM as % specific lysis of at least 3 independent experiments, each performed in triplicates. Data were analyzed by two-way ANOVA, and significant differences between treated and non-treated cells (*, p ≤ 0.05) are indicated.

### Supplementary Figure 5


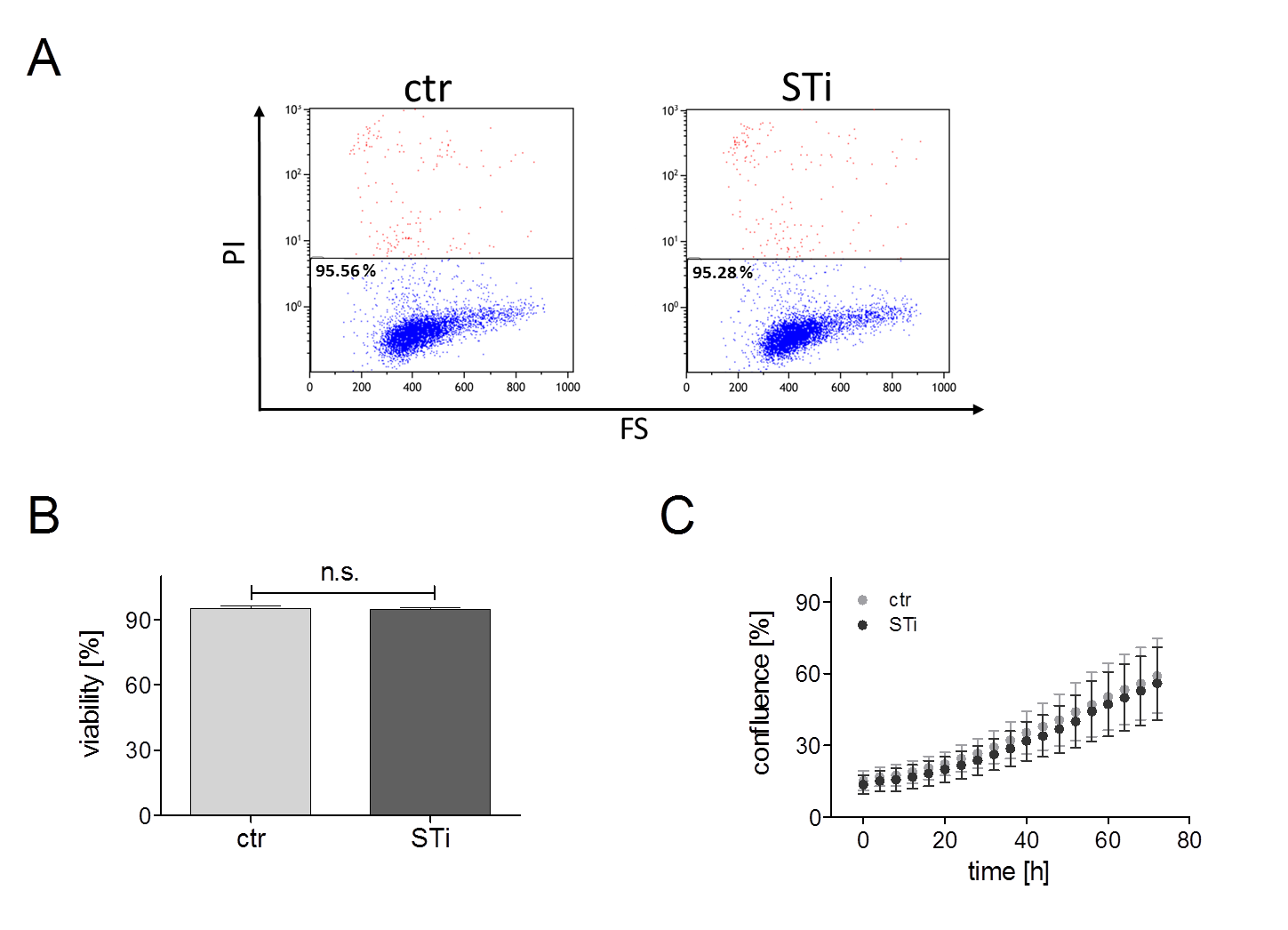


**Figure S5: Treatment of tumor cells with a sialyltransferase inhibitor (STi) does not interfere with cell viability and proliferation. (A)** A representative dot plot of propidium iodide (PI) staining of MDA-MB-468 cells. **(B)** Bar plots showing viability measured by PI staining after STi treatment compared to untreated cells of three biological experiments. **(C)** Tumor cell confluency measured by real-time microscopy of untreated versus with STi (100 µM, 72h) treated MDA-MB-468 cells. Statistical analyses were performed with parametric paired student t-test (p < 0.05) between treated and untreated cells. n.s., not significant.

### Supplementary Figure 6


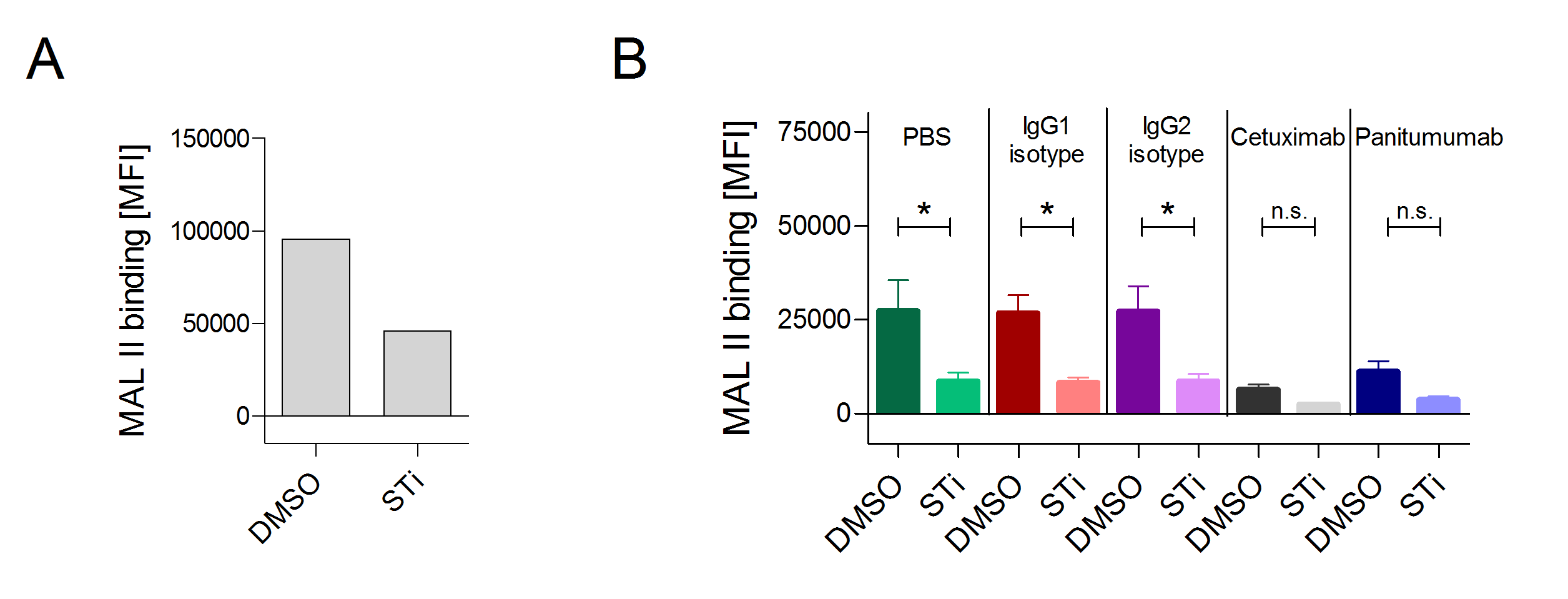


**Figure S6: MAL II binding after treatment of tumor cells with sialyltransferase inhibitor (STi) *in vitro* and *ex vivo*. (A)** Sialoglycans expression on MDA-MB-468 cells after sialyltransferase (STi) treatment prior to *in vivo* injection. Binding of MAL II (5 µg/ml) on MDA-MB-468 cells is reduced after treatment with STi *in vitro*. **(B)** MAL II binding (5 µg/ml) to DMSO or STi treated MDA-MB-468 breast cancer cells *ex vivo* from the differently treated mice groups.

### Supplementary Figure 7


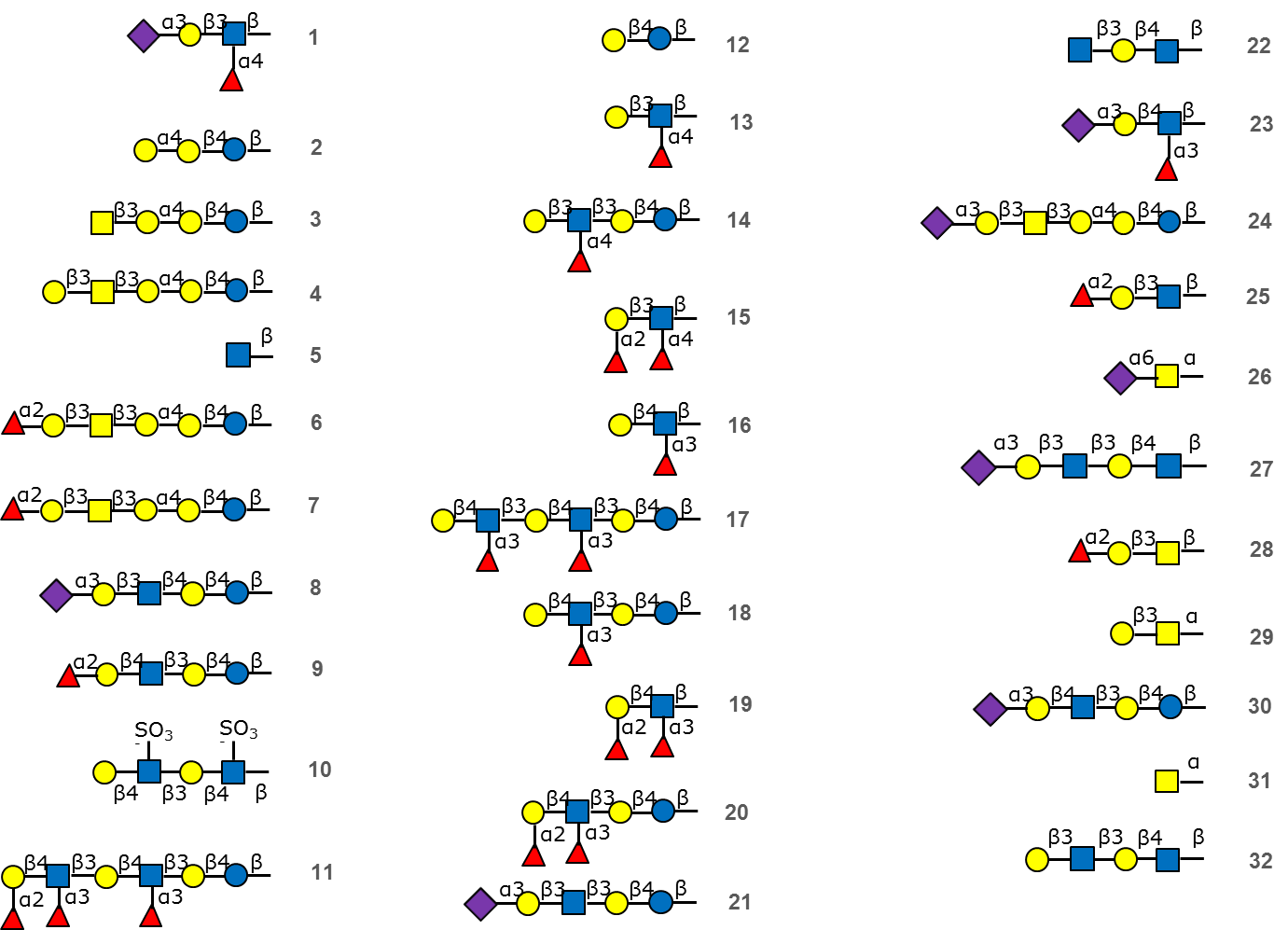


**Figure S7: Glycan array.** Synthetic glycans which were used to test for Siglec-9-Fc binding to identify potential tumor associated carbohydrate antigens (TACAs).

## Supplementary Tables

### Supplementary Table 1

| **Number** | **Glycan** | **Trivial name** |
| --- | --- | --- |
| 1 | Neu5Ac(α2-3)Gal(β1-3)[Fuc(α1-4)]GlcNAc(β1-1)-Linker | CA19-9/sLeA |
| 2 | Gal(α1-4)Gal(β1-4)Glc(β1-1)-Linker | Gb3 |
| 3 | GalNAc(β1-3)Gal(α1-4)Gal(β1-4)Glc(β1-1)-Linker | Gb4 |
| 4 | Gal(β1-3)GalNAc(β1-3)Gal(α1-4)Gal(β1-4)Glc(β1-1)-Linker | Gb5 |
| 5 | GlcNAc(β1-1)-Linker | - |
| 6 | Fuc(α1-2)Gal(β1-3)GalNAc(β1-3)Gal(α1-4)Gal(β1-4)Glc(β1-1)-Linker | GloboH |
| 7 | Fuc(α1-2)Gal(β1-3)GalNAc(β1-3)Gal(α1-4)Gal(β1-4)Glc(β1-1)-Linker | GloboH |
| 8 | Neu5Ac(α2-3)Gal(β1-3)GlcNAc(β1-4) Gal(β1-4)Glc(β1-1)-Linker | GM1b |
| 9 | Fuc(α1-2)Gal(β1-4)GlcNAc(β1-3)Gal(β1-4)Glc(β1-1)-Linker | H-Antigen II |
| 10 | Gal(β1-4)[6S]GlcNAc(β1-3) Gal(β1-4)[6S]GlcNAc(β1-1)-Linker | - |
| 11 | Fuc(α1-2)Gal(β1-4)[Fuc(α1-3)]GlcNAc(β1-3)Gal(β1-4)[Fuc(α1-3)]GlcNAc(β1-3)Gal(β1-4)Glc(β1-1)-Linker | LeY-LeX |
| 12 | Gal(β1-4)Glc(β1-1)-Linker | Lactose |
| 13 | Gal(β1-3)[Fuc(α1-4)]GlcNAc(β1-1)-Linker | LeA |
| 14 | Gal(β1-3)[Fuc(α1-4)]GlcNAc(β1-3)Gal(β1-4)Glc(β1-1)-Linker | LeA |
| 15 | Fuc(α1-2)Gal(β1-3)[Fuc(α1-4)]GlcNAc(β1-1)-Linker | LeB |
| 16 | Gal(β1-4)[Fuc(α1-3)]GlcNAc(β1-1)-Linker | LeX |
| 17 | Gal(β1-4)[Fuc(α1-3)]GlcNAc(β1-3)Gal(β1-4)[Fuc(α1-3)]GlcNAc(β1-3)Gal(β1-4)Glc(β1-1)-Linker | LeX-LeX |
| 18 | Gal(β1-4)[Fuc(α1-3)]GlcNAc(β1-3)Gal(β1-4)Glc(β1-1)-Linker | LeX |
| 19 | Fuc(α1-2)Gal(β1-4)[Fuc(α1-3)]GlcNAc(β1-1)-Linker | LeY |
| 20 | Fuc(α1-2)Gal(β1-4)[Fuc(α1-3)]GlcNAc(β1-3)Gal(β1-4)Glc(β1-1)-Linker | LeY |
| 21 | Neu5Ac(α2-3)Gal(β1-3)GlcNAc(β1-3) Gal(β1-4)Glc(β1-1)-Linker | LSTa |
| 22 | GlcNAc(β1-3) Gal(β1-4)GlcNAc(β1-1)-Linker | - |
| 23 | Neu5Ac(α2-3)Gal(β1-4)[Fuc(α1-3)]GlcNAc(β1-1)-Linker | sLeX |
| 24 | Neu5Ac(α2-3)Gal(β1-3)GalNAc(β1-3)Gal(α1-4)Gal(β1-4)Glc(β1-1)-Linker | SSEA4 |
| 25 | Fuc(α1-2)Gal(β1-3)GlcNAc (β1-1)-Linker | SSEA5 |
| 26 | Neu5Ac(α2-6) GalNAc(α 1-1)-Linker | sTn |
| 27 | Neu5Ac(α2-3)Gal(β1-3)GlcNAc(β1-3) Gal(β1-4)GlcNAc(β1-1)-Linker | sTRA |
| 28 | Fuc(α1-2)Gal(β1-3)GalNAc(β1-1)-Linker | - |
| 29 | Gal(β1-3)GalNAc(α 1-1)-Linker | Tf |
| 30 | Neu5Ac(α2-3)Gal(β1-4)GlcNAc(β1-3) Gal(β1-4)Glc(β1-1)-Linker | - |
| 31 | GalNAc(α 1-1)-Linker | Tn |
| 32 | Gal(β1-3)GlcNAc(β1-3) Gal(β1-4)GlcNAc(β1-1)-Linker | TRA |

**Table S1: List of synthetic glycans.** Structures and names of the synthetic glycans used to test for Siglec-9-Fc binding.
